# Genome-wide analysis of WRKY transcription factors in white pear (*Pyrus bretschneideri*) reveals evolution and patterns under drought stress

**DOI:** 10.1186/s12864-015-2233-6

**Published:** 2015-12-24

**Authors:** Xiaosan Huang, Kongqing Li, Xiaoyong Xu, Zhenghong Yao, Cong Jin, Shaoling Zhang

**Affiliations:** College of Horticulture, State Key Laboratory of Crop Genetics and Germplasm Enhancement, Nanjing Agricultural University, Nanjing, 210095 China; College of Rural Development, Nanjing Agricultural University, Nanjing, 210095 China; School of Horticulture and Plant Protection, Yangzhou University, Yangzhou, 225009 China

**Keywords:** Pear, WRKY transcription factor, Drought stress

## Abstract

**Background:**

WRKY transcription factors (TFs) constitute one of the largest protein families in higher plants, and its members contain one or two conserved WRKY domains, about 60 amino acid residues with the WRKYGQK sequence followed by a C_2_H_2_ or C_2_HC zinc finger motif. WRKY proteins play significant roles in plant development, and in responses to biotic and abiotic stresses. Pear (*Pyrus bretschneideri*) is one of the most important fruit crops in the world and is frequently threatened by abiotic stress, such as drought, affecting growth, development and productivity. Although the pear genome sequence has been released, little is known about the WRKY TFs in pear, especially in respond to drought stress at the genome-wide level.

**Results:**

We identified a total of 103 WRKY TFs in the pear genome. Based on the structural features of WRKY proteins and topology of the phylogenetic tree, the pear WRKY (PbWRKY) family was classified into seven groups (Groups 1, 2a–e, and 3). The microsyteny analysis indicated that 33 (32 %) *PbWRKY* genes were tandemly duplicated and 57 genes (55.3 %) were segmentally duplicated. RNA-seq experiment data and quantitative real-time reverse transcription PCR revealed that *PbWRKY* genes in different groups were induced by drought stress, and Group 2a and 3 were mainly involved in the biological pathways in response to drought stress. Furthermore, adaptive evolution analysis detected a significant positive selection for Pbr001425 in Group 3, and its expression pattern differed from that of other members in this group. The present study provides a solid foundation for further functional dissection and molecular evolution of *WRKY* TFs in pear, especially for improving the water-deficient resistance of pear through manipulation of the *PbWRKYs*.

**Electronic supplementary material:**

The online version of this article (doi:10.1186/s12864-015-2233-6) contains supplementary material, which is available to authorized users.

## Background

Transcriptional regulation of gene expression is one of the most important regulatory mechanisms and transcription factors (TFs) mediate transcriptional regulation in response to developmental and environmental changes in plants. WRKY family is one of the largest TF families in higher plants, but is absent from animals. Since the discovery of the WRKY domain with DNA-binding capability [1], members of the WRKY protein family have been found to have an ever increasing number of functions in essential physiological and developmental processes in plants [2]. WRKY proteins contain either one or two WRKY domains. The WRKY domain contains approximately 60 amino acids with the conserved amino acid sequence WRKYGQK at its N-terminus and a zinc finger motif, C_2_H_2_ (C–X_4–5_–C–X_22–23_–H–X–H) or C_2_HC (C–X_7_–C–X_23_–H–X–C), at the C-terminal region [3]. The WRKY family can be classified on the basis of both the number of WRKY domains and the features of their zinc-finger motif. WRKY proteins with two WRKY domains belong to Group 1, whereas proteins with one WRKY domain belong to Group 2 or 3. Generally, the WRKY domains of members of Groups 1 and 2 members have the same type of finger motif, C_2_H_2_. The single finger motif of Group 3 is distinct from that of members of Groups 1 and 2. Instead of a C_2_H_2_ pattern, their WRKY domains contain a C_2_HC motif. However, the WRKYGQK amino acid sequence of all members in three groups forms a β-strand that binds sequence-specifically to the DNA sequence motif (T)(T)TGAC(C/T), which is known as the W box [4].

Experimental evidences has shown that plant WRKY proteins are involved in responses to biotic and abiotic stresses, and in developmental processes [2]. WRKY proteins play an important role in plant defense against biotic stresses, such as bacterial, fungal, and viral pathogens [5, 6]. WRKY proteins are also involved in plant-specific processes, such as trichome development [7], embryogenesis [8], seed development [9], dormancy [10], and senescence [11]. They are also key components in some signal transduction processes mediated by plant hormones such as gibberellic acid [12], abscisic acid (ABA) [13], and salicylic acid [14]. It is also well documented that WRKY proteins are involved in responses to various abiotic stresses, such as salinity, drought, and cold [15, 16]. Accumulating evidences shows that WRKY genes play an important role in responses to drought stress. ABO3, a WRKY TF, mediates plant responses to ABA and drought tolerance in *Arabidopsis* [15]; 10 *TaWRKY* genes responsive to drought stress were identified in an RNA-seq experiment [17]; and 42 *OsWRKY* genes were inducible under drought treatment [18].

Pear (*Pyrus bretschneideri*) is one of the most important fruit crops in the world. In the field, pear frequently experiences abiotic stress, such as drought, which is a key factor affecting growth, development and productivity. Recently, the complete genome of pear was sequenced by the Centre of Pear Engineering Technology Research, Nanjing Agricultural University (http://peargenome.njau.edu.cn/) [19]. This completed genome provides an opportunity to better understand the evolution and function of the WRKY family at the whole-genome level. Many WRKY proteins have been reported to be involved in responses to drought stress and so our initial interest was in the drought-related *WRKY* genes in pear. In this study, we identified 103 pear *WRKY* (*PbWRKY*) genes from the pear genomic sequence and carried out phylogenetic analysis to determine the relationships among these pear genes. Analysis of protein motifs and intron/exon structures provided support for the classification of the WRKY family. Furthermore, we identified the duplication events that likely contributed to the expansion of the WRKY family. In addition, RNA-seq data showed the expression patterns of *PbWRKYs* in different water-deficient stress, and subsequent quantitative real-time PCR (qRT-PCR) analysis indicated that two groups (Groups 2a and 3) of this family responded to drought stress. Then, to examine the driving force for the evolution of function for genes in Groups 2a and 3, we further analyzed adaptive evolution at the amino acid level. Our systematic analysis provided a foundation for further functional dissection and molecular evolution of *WRKY* genes in pear, especially for improving the drought resistance of pear and through the manipulation of *PbWRKYs*.

## Results

### The PbWRKY protein family consists of at least 103 members

**Fig. 1:**
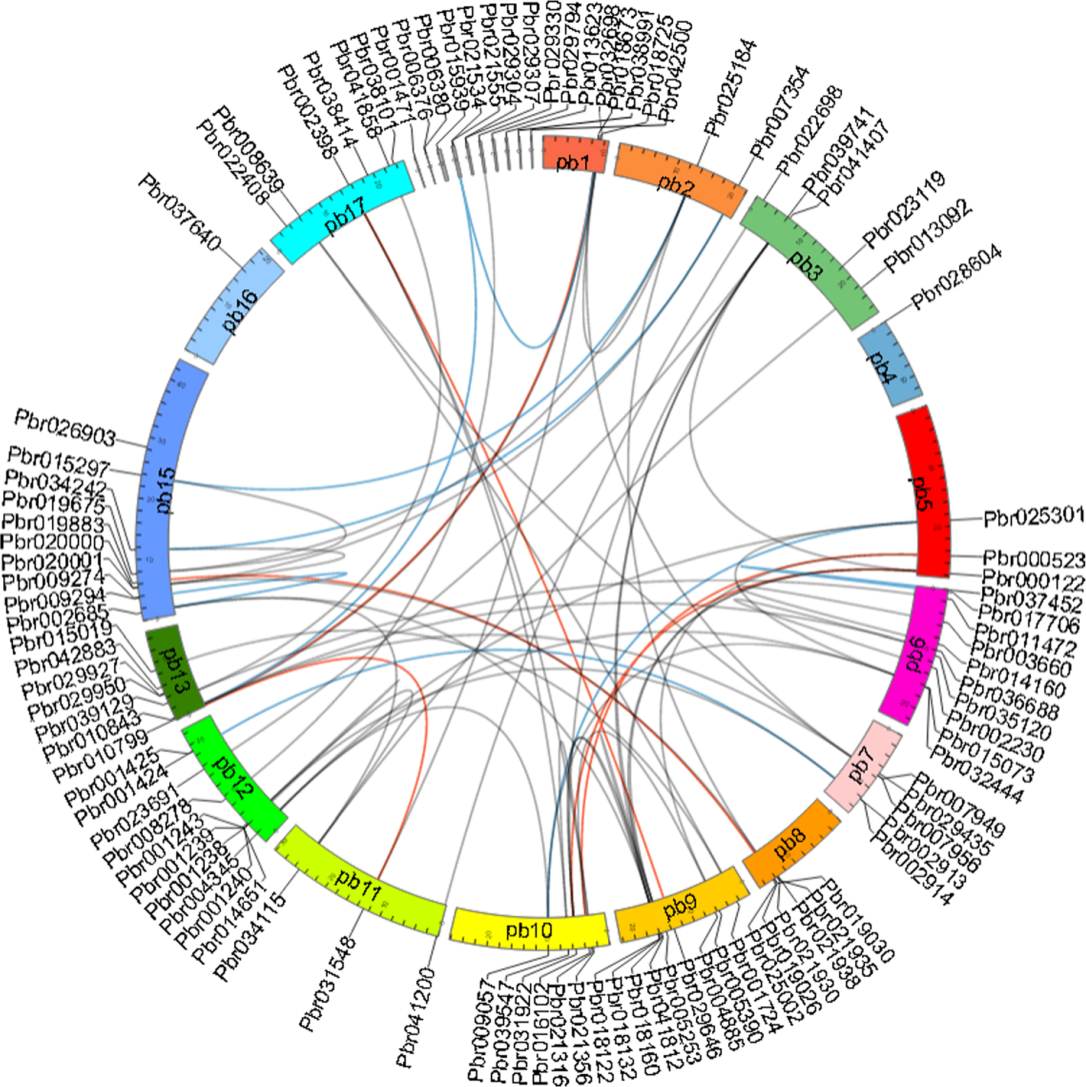
Localization and duplication of the *WRKY* genes in the pear genome. Circular visualization of the 103 *WRKY* genes was mapped on the different chromosomes in the pear genome using Circos software. Chromosome number is indicated on the chromosome. The microsynteny between each pair of *WRKY* genes were detected by using the MicroSyn software. The genes with synteny relationship are linked by lines. Red link: ≥30 anchors in a synteny block, blue link: 20–30 anchors, green link: 10–20 anchors, gray link: 5–10 anchors

To obtain sequences of *WRKY* genes in the pear genome, we used a HMMER-BLASTP-InterProScan strategy to search for genes encoding proteins containing the Pfam PF03106 domain. In total, 103 *PbWRKY* genes were identified (Additional file 1). Of the 103 *PbWRKY* genes, 87 were mapped on all pear chromosomes except chromosome 14, and 16 *PbWRKY* genes were located on scaffold contigs (Fig. 1). Among the other 87 *PbWRKY* genes, there were 10 respectively situated on chromosomes 6, 12 and 15; nine on chromosome 9; seven on chromosome 13; six each on chromosomes 7 and 8; five respectively on chromosomes 3, 10 and 17; three respectively on chromosomes 1, 5 and 11; two on chromosomes 2; and only one gene respectively on chromosomes 4 and 16.

### Phylogenetic analysis of pear *WRKY* genes

**Fig. 2:**
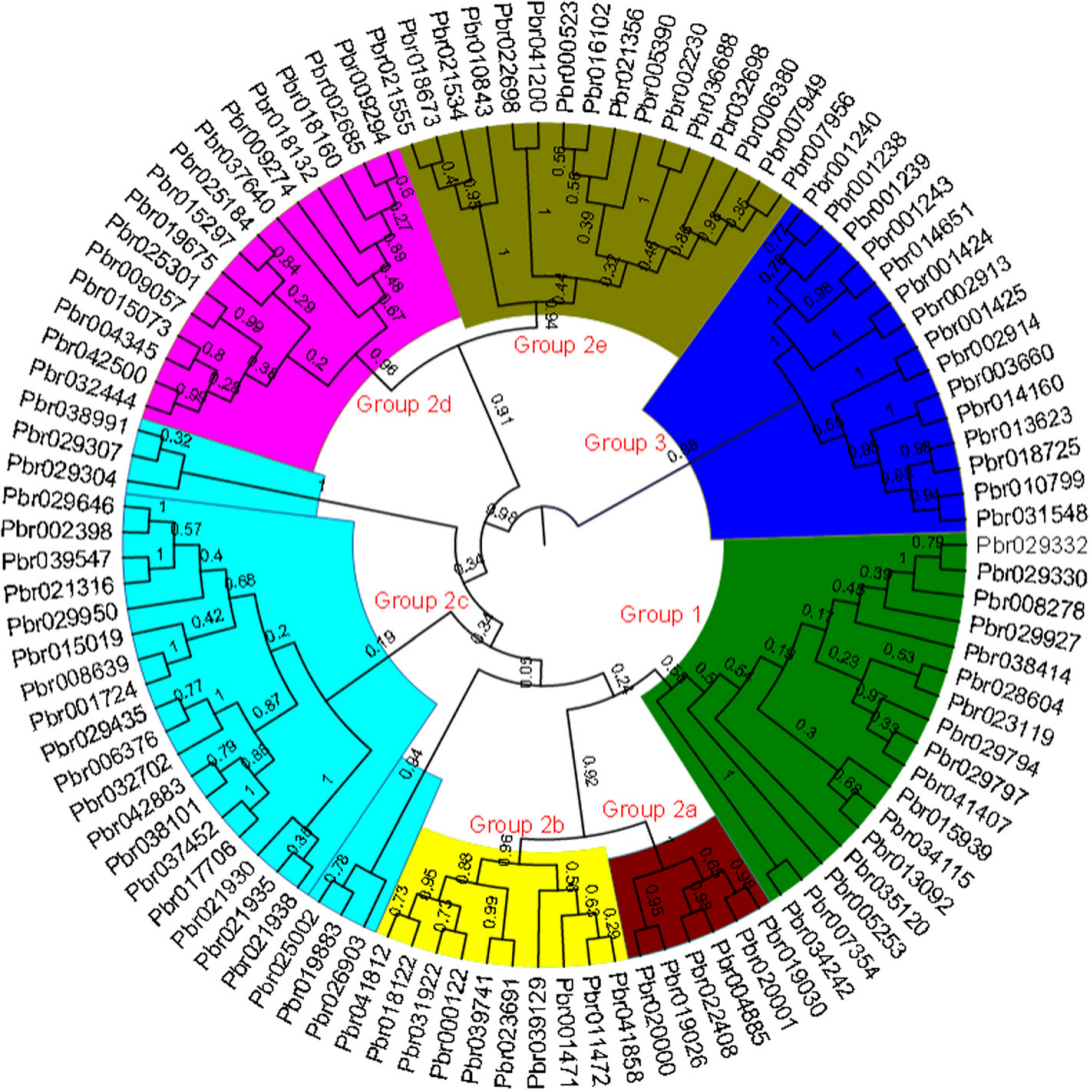
Phylogenetic trees of *WRKY* genes in pear. The un-rooted phylogenetic tree of WRKY domains was constructed with MEGA5.1 program with the NJ method. The numbers beside the branches represent bootstrap values based on 1000 replications. The name of groups (1, 2a-e, and 3) are shown at the inside of the circle. The groups of genes are shown in different colors

**Fig. 3:**
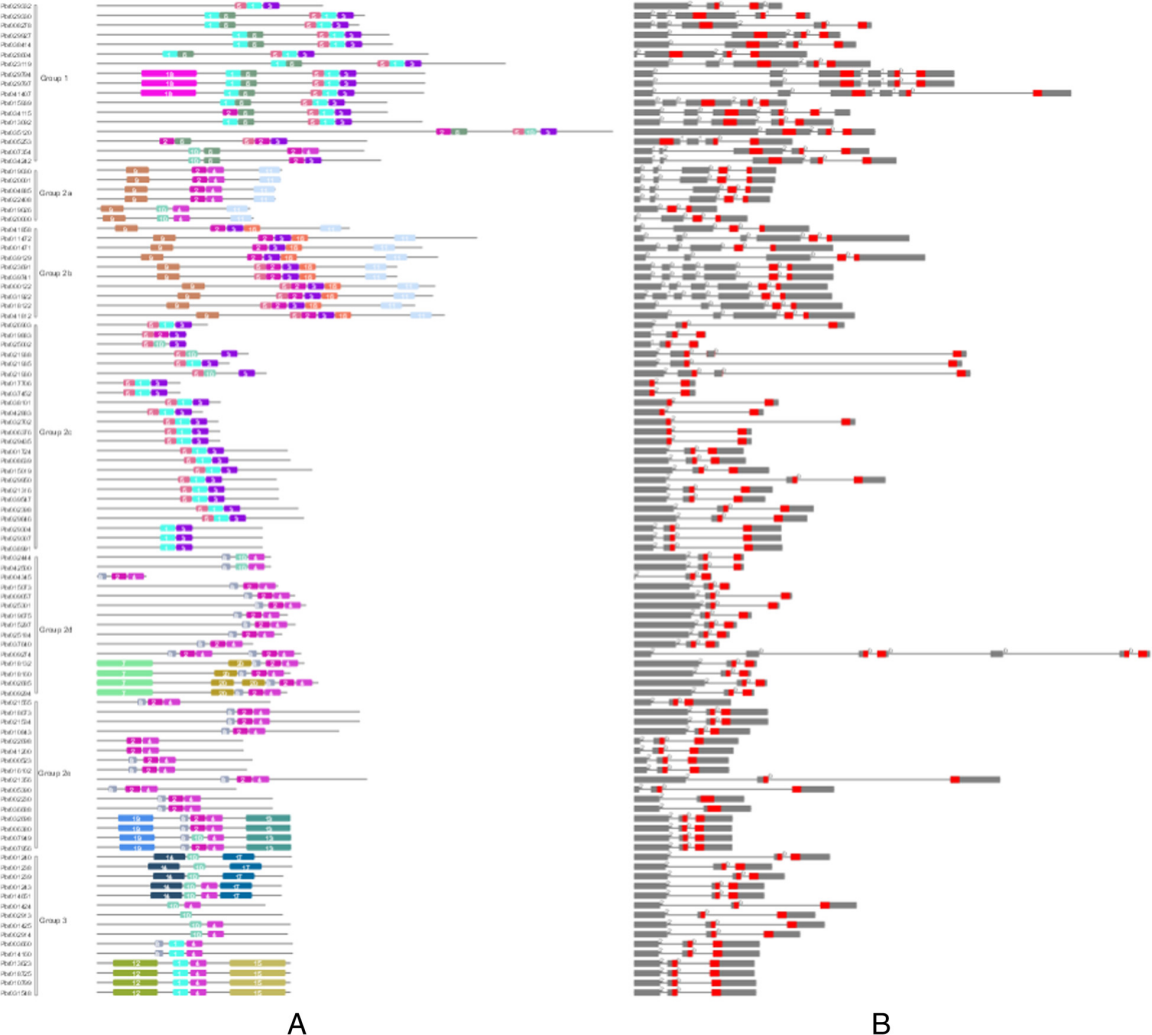
Schematic representations of the conserved motifs and exon–intron compositions. Names of genes are indicated on the left. **a** Conserved motifs in WRKY proteins. Different motifs are highlighted with different colored boxes with numbers 1 to 20. Lines represent protein regions without detected motif. **b** Exon–intron compositions in *WRKY* gene. Exons, represented by gray or red boxes, are drawn to scale. Dashed lines connecting two exons represent an intron. WRKY domain is marked in red

In previous studies, WRKY TFs were classified into seven groups based on their number of WRKY domains and the pattern of their zinc finger motif [3]. Group 1 contains two WRKY domains (N-terminal and C-terminal), including a C_2_H_2_ motif, whereas Group 2a–e and 3 have only one domain. Group 3 has a distinct zinc finger motif, C_2_HC. To investigate the phylogenetic relationships of the *WRKY* genes in pear, we first constructed an un-rooted phylogenetic tree of 103 *PbWRKY* genes from the multiple sequence alignment of their WRKY domains. Three methods, Neighbor-Joining (NJ), Maximum Likelihood (ML), and Maximum Parsimony (MP) generated nearly identical topologies of phylogenetic trees (Additional files 2 and 3), although the support values at some inter nodes are different. Therefore, only the NJ tree was used for further analysis (Fig. 2). To better separate the groups and examine the evolutionary relationships of *PbWRKY* genes, we considered the tree topology , as well as the conserved sequence feature (i.e. number of WRKY domains and the pattern of zinc finger motif). The *PbWRKY* genes were first divided into three distinct groups: Groups 1, 2 and 3. This classification was consistent with results of previous studies. Group 1 (17 genes) contained two WRKY domains and was distinctly separated from other groups. However, Pbr029332 with only one WRKY domain was clustered with C-terminal WRKY domains of Group 1. Pbr029332 may have lost the N-terminal WRKY domain during evolution, or the N-terminal part of this gene was annotated incorrectly. Group 1 *PbWRKY* genes had a C_2_H_2_-type zinc-finger motif in the C-terminal WRKY domain. Group 2 contained 71 *PbWRKY*s, which possessed a single WRKY domain and a C_2_H_2_-type zinc-finger motif. Group 3 comprised 15 *PbWRKY* genes with a single WRKY domain. The C_2_HC zinc-finger structure in this group differed from those in other groups. Additionally, PbWRKY proteins in Group 2 had diverse sequences (Fig. 3). The structure and phylogenetic tree clearly indicated that Group 2 proteins could be divided into five distinct subgroups: a–e.

### Conserved structural features of PbWRKY proteins

The most prominent feature of proteins in WRKY TFs is the WRKY domain, which preferentially binds to the promoter of their downstream target genes on a specific cis-element (e.g. W-box). We surveyed up to top 20 motifs in the 103 PbWRKY proteins using MEME (Fig. 3 and Additional file 4). Motifs 1, 2 and 6 were the conserved motifs at the N-terminus of the WRKY domain, while motifs 3–5 represented the zinc-finger motif at the C-terminus. The distribution of motifs outside the WRKY domain was highly conserved within groups. For example, motifs 9 and 12 only appeared in Groups 2a and 2b; and motifs 10 and 37 appeared exclusively in Group 2d.

### Structure of *PbWRKY* genes

Since the intron/exon organizations and intron types and numbers are typical imprints of evolution within some gene families, we examined the *PbWRKY* gene structures to gain further insight into their evolutionary events. All *WRKY* genes in Groups 2 and 3 contained one intron in their WRKY domains except for two genes in Group 2e (Fig. 3). In addition, the exon/intron structures outside the WRKY domain were highly conserved within groups. Each group of *PbWRKY* genes mostly shared the same intron/exon structural pattern. One intron with phase 2 in the N-terminal existed in Groups 2d, 2e and 3; and there were three or four introns with phase 0 in Groups 2a and 2b.

### Whole-genome duplication analysis of *PbWRKY* genes

It is thought that gene families evolved from a process of genome-wide duplication, segmental duplication and tandem duplication accompanied by post-duplication diversification [20–22]. Duplication events can result in a clustered occurrence of family members through tandem amplification, or a scattered occurrence through segmental duplication of chromosomal regions [20–22]. In this analysis, we focused on the tandem and segmental duplication modes of *WRKY* TFs in the whole pear genome. To identify the amplification patterns of the *WRKY* TFs, we first detected the existence of tandem duplications. We defined tandem duplication as one falling within 10 neighbors of another on genomic regions. Of the 103 *PbWRKY* genes, 33 (32 %) genes formed 15 tandemly duplicated clusters. The information concerning tandemly duplicated *WRKY* genes in pear is listed in Table 1, including four genes in Group 1, four in Group 2a, seven in Group 2c, six in Group 2d, two in Group 2b and seven in Group 3. No putative tandemly duplicated genes were found in Group 2d. To detect the segmental duplication events between two members in a gene family, the DNA sequences containing their neighboring genes were considered. Conserved, flanking collinear homologous gene pairs between the two genomic regions were searched for microsynteny to determine the segmental duplication events, controlled by a statistical distance function [23]. There were 61 pairs with collinear relationships detected and 57 genes (55.3 %) were involved in segment duplication (Table 2), suggesting that segmental duplication contributed to the expansion of the *WRKY* gene family in the pear genome. Taken together, tandem and segmental duplication events were involved in the expansion of the WRKY family in the pear genome.

**Table 1:** Genes involved in tandem duplication

Tandem duplicated genes	Group	Chromosome
Pbr029794, Pbr029797	1	scaffold503.0
Pbr029330, Pbr029332	1	scaffold491.0
Pbr019026, Pbr019030	2a	Chr8
Pbr020001, Pbr020000	2a	Chr15
Pbr029304, Pbr029307	2c	scaffold490.0
Pbr021938, Pbr021935, Pbr021930	2c	Chr8
Pbr006376, Pbr006380	2c	scaffold1326.0
Pbr032698, Pbr032702	2c	scaffold591.0
Pbr015073, Pbr032444	2d	Chr6
Pbr018132, Pbr018122	2d	Chr9
Pbr009294, Pbr009274	2d	Chr15
Pbr007956, Pbr007949	2e	Chr7
Pbr002913, Pbr002914	3	Chr7
Pbr001238, Pbr001243, Pbr001239, Pbr001240	3	Chr12
Pbr001424, Pbr001425	3	Chr12

**Table 2:** Synteny related to *WRKY* genes in pear

Gene pairs	Anchors	E-value	Mean Ks	SD	Duplicated type
Pbr002398 vs. Pbr029646	33	8.58E-49	0.19	0.09	segmental
Pbr000122 vs. Pbr031922	33	3.71E-49	0.18	0.08	segmental
Pbr031548 vs. Pbr010799	33	9.59E-56	0.03	0.03	segmental
Pbr000523 vs. Pbr016102	32	1.77E-48	0.19	0.05	segmental
Pbr025002 vs. Pbr019883	32	2.91E-46	0.25	0.23	segmental
Pbr018673 vs. Pbr010843	31	9.14E-46	0.26	0.35	segmental
Pbr037452 vs. Pbr017706	29	6.97E-47	0.03	0.09	segmental
Pbr007354 vs. Pbr034242	29	5.27E-48	0.21	0.1	segmental
Pbr025301 vs. Pbr009057	28	5.33E-38	0.18	0.06	segmental
Pbr018673 vs. Pbr021534	27	5.31E-41	0.05	0.05	segmental
Pbr001424 vs. Pbr002913	26	1.11E-41	0.22	0.09	segmental
Pbr002685 vs. Pbr009294	26	1.67E-45	0.01	0.01	segmental
Pbr015297 vs. Pbr025184	24	8.01E-30	0.27	0.34	segmental
Pbr010843 vs. Pbr021534	22	4.27E-33	0.19	0.05	segmental
Pbr010799 vs. Pbr018725	19	9.26E-29	0.28	0.35	segmental
Pbr039547 vs. Pbr021316	18	1.25E-33	0.03	0.12	segmental
Pbr008639 vs. Pbr001724	15	6.05E-20	0.23	0.25	segmental
Pbr013092 vs. Pbr034115	14	6.10E-16	0.55	0.92	segmental
Pbr008278 vs. Pbr029330	14	2.54E-18	0.23	0.15	segmental
Pbr031548 vs. Pbr018725	13	1.72E-22	0.2	0.07	segmental
Pbr015073 vs. Pbr004345	12	4.56E-20	0.25	0.28	segmental
Pbr011472 vs. Pbr039129	12	9.50E-12	1.38	0.42	segmental
Pbr041812 vs. Pbr018132	12	5.92E-19	0.09	0.28	segmental
Pbr019675 vs. Pbr025184	11	2.87E-10	1.27	0.48	segmental
Pbr036688 vs. Pbr002230	11	3.00E-20	0.05	0.13	segmental
Pbr019675 vs. Pbr015297	10	6.23E-10	1.36	0.75	segmental
Pbr000122 vs. Pbr041812	10	4.80E-14	1.25	0.62	segmental
Pbr039741 vs. Pbr031922	10	2.94E-15	1.49	0.95	segmental
Pbr022698 vs. Pbr041200	10	1.01E-18	0.19	0.06	segmental
Pbr000122 vs. Pbr039741	9	2.71E-14	1.57	0.9	segmental
Pbr031922 vs. Pbr018132	9	2.06E-10	1.34	0.43	segmental
Pbr019026 vs. Pbr020001	9	9.84E-16	0.35	0.52	segmental
Pbr023691 vs. Pbr039741	8	4.43E-14	0.1	0.27	segmental
Pbr000122 vs. Pbr018132	8	1.57E-09	1.57	0.43	segmental
Pbr025301 vs. Pbr004345	8	1.33E-08	1.04	0.63	segmental
Pbr041812 vs. Pbr031922	8	1.14E-11	1.36	0.35	segmental
Pbr034242 vs. Pbr020001	8	3.02E-06	1.49	0.42	segmental
Pbr002398 vs. Pbr039547	7	2.62E-06	1.57	0.46	segmental
Pbr001238 vs. Pbr014651	7	2.56E-11	0.18	0.05	segmental
Pbr039741 vs. Pbr041812	7	1.94E-10	1.27	0.25	segmental
Pbr039741 vs. Pbr018132	7	1.79E-08	1.38	0.25	segmental
Pbr041858 vs. Pbr039129	7	7.25E-09	1.33	0.78	segmental
Pbr005390 vs. Pbr000523	6	5.22E-08	1.59	0.56	segmental
Pbr004345 vs. Pbr009057	6	2.11E-07	1.11	0.47	segmental
Pbr002685 vs. Pbr018132	6	5.22E-08	0.2	0.08	segmental
Pbr018673 vs. Pbr007956	6	1.91E-06	1.15	0.15	segmental
Pbr003660 vs. Pbr014160	6	2.09E-12	0.02	0.03	segmental
Pbr007354 vs. Pbr020001	6	2.82E-05	1.57	0.44	segmental
Pbr034115 vs. Pbr014651	6	5.51E-06	1.43	0.5	segmental
Pbr002398 vs. Pbr021316	5	1.44E-05	1.66	0.5	segmental
Pbr015073 vs. Pbr025301	5	3.91E-07	1.01	0.21	segmental
Pbr015073 vs. Pbr009057	5	3.02E-05	1.18	0.38	segmental
Pbr002913 vs. Pbr014651	5	5.94E-06	1.27	0.45	segmental
Pbr036688 vs. Pbr010843	5	1.38E-04	1.51	0.26	segmental
Pbr005390 vs. Pbr016102	5	1.64E-06	1.9	0.56	segmental
Pbr002685 vs. Pbr034115	5	1.44E-03	1.36	0.71	segmental
Pbr008639 vs. Pbr007956	5	4.32E-04	1.72	0.86	segmental
Pbr001724 vs. Pbr015019	5	2.97E-05	1.51	0.42	segmental
Pbr007956 vs. Pbr010843	5	1.13E-03	1.11	0.33	segmental
Pbr025184 vs. Pbr019026	5	5.60E-03	1.59	0.49	segmental
Pbr025184 vs. Pbr018725	5	3.98E-06	1.51	0.79	segmental

### Expression of *PbWRKY* genes under drought stress

**Fig. 4:**
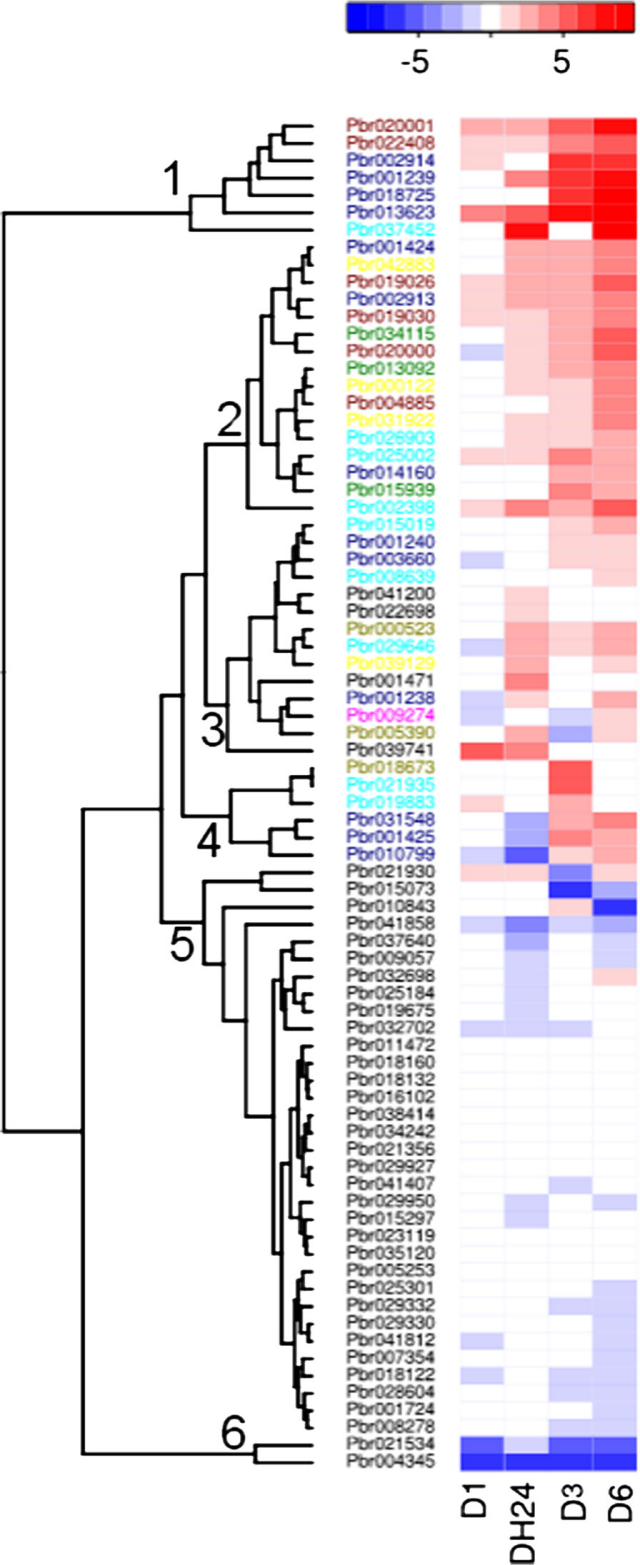
Heat map of RNA-seq expression of *PbWRKY* gene in response to drought stress. Color scale of the dendrogram represents log2 ratio value of treated sample to control sample. D1, D3, D6 and D24, dehydrated for 1, 3 and 6 h in an ambient environment and recovered for 24 h in water, respectively. The colors of genes in Cluster 1–4 represent their groups in the phylogenetic tree in Fig. 2

Some evidence has suggested that PbWRKY proteins are involved in signaling and responses to abiotic stimuli [15, 18], such as drought stress, but limited information is available on involvement of WRKY TFs in drought stress response of pear. In this study, RNA-seq data for short-term dehydration stress on pear seedling treatment were from parallel work (paper in preparation) aiming to study the response to water-deficiency stresses of pear. The heatmap was divided into six clusters (Fig. 4). Cluster 1 contained seven (6.8 %) detectable *PbWRKY* genes, which were significantly up-regulated by drought treatment at 3 and 6 h, except for Pbr037452. Cluster 2 contained 16 genes, which were also highly induced at 3 and 6 h after drought treatment, but their relative expression levels (compared with control) were lower than for genes in Cluster 1. In Clusters 3 (14 genes) and 4 (six genes), most genes were up-regulated after 3 and/or 6 h of drought treatment; however, some genes (Pbr041200, Pbr001471 and Pbr039741) were not induced by drought stress. Genes in Clusters 5 and 6 were down-regulated or not induced by drought treatment. Overall, 44 *PbWRKY* genes were up-regulated at least two-fold after drought treatment relative to controls and were within the range of 2–1024 fold; and 19 *PbWRKY* genes were down-regulated at least two-fold after drought treatment relative to controls within the range of 2–64 fold. We focused on the up-regulated genes of Clusters 1, 2 and 3 and the expression patterns in phylogenetic groups were surveyed.

We found that all six genes in Group 2a, 13 (86.7 %) in Group 3, four (40.0 %) in Group 2b, nine (37.5 %) in Group 2c, three (18.8 %) in Group 2e, three (17.6 %) in Group 1 and one gene (6.7 %) in Group 2d were up-regulated in response to drought stress. Genes in Cluster 1 were more significantly up-regulated than genes in the other clusters, and most genes in Cluster 1 belonged to Groups 2b and 3. This result suggests that *PbWRKY* genes in different groups were induced by water deficiency, and Groups 2a and 3 were mainly involved in the biological pathways responding to drought stress.

Orthologous genes are homologous genes that have diverged after a speciation event. Orthologous genes are generally assumed to retain equivalent functions in different organisms and to share other key properties. In this type of homologous gene, the ancestral gene and its function is maintained through a speciation event, although variations may arise within the gene after the point at which the species diverged [24]. In the published literature, 13 WRKY TFs have been shown to be involved in drought, salt and osmotic stresses [15, 25–34]. Nine of the 13 WRKY TFs located in eight orthologous groups were identified using InParanoid [35]. Six of the eight orthologous groups had 14 stress-responsive PbWRKY TFs (Additional file 5). These 14 stress-responsive PbWRKY TFs may retain equivalent functions to those in *Arabidopsis*. However, we found four WRKY TFs that were not located in orthologous groups, indicating they did not have equivalent PbWRKY TFs. Taken together, these results indicate that the functions of PbWRKY TFs were largely conserved.

In plants, transcriptional regulation is mediated by a large number (>1500) of TFs controlling the expression of tens or hundreds of target genes in various, sometimes intertwined, signal transduction cascades [36]. As TFs, WRKY TFs can bind to cis-elements to control the expression of tens or hundreds of target genes in plants. To understand the function of WRKY TFs at the system level, we investigated the gene co-expression clusters that had drought-responsive WRKY TFs. Nine co-expression networks were found to have drought-responsive WRKY TFs (Additional file 6). There were 1–22 drought-responsive WRKY TFs. Co-expression gene Cluster 3 had the greatest number of WRKY TFs (i.e. 22). Gene ontology (GO) analysis showed that genes in Cluster 3 were enriched in GO terms of regulation of macromolecule biosynthetic process, protein modification process, response to biotic stimulus, biological regulation, macromolecule modification, response to water stress, aromatic amino acid family biosynthetic process, chorismate metabolic process, phosphate metabolic process, phosphorus metabolic process, secretion, and secretion by cell. Furthermore, Kyoto Encyclopedia of Genes and Genomes (KEGG) pathway enrichment analysis showed that genes located in co-expression gene Cluster 3 were enriched in plant hormone signal transduction and circadian entrainment. These two pathways are well-known to be related to drought stress [37, 38].

**Fig. 5:**
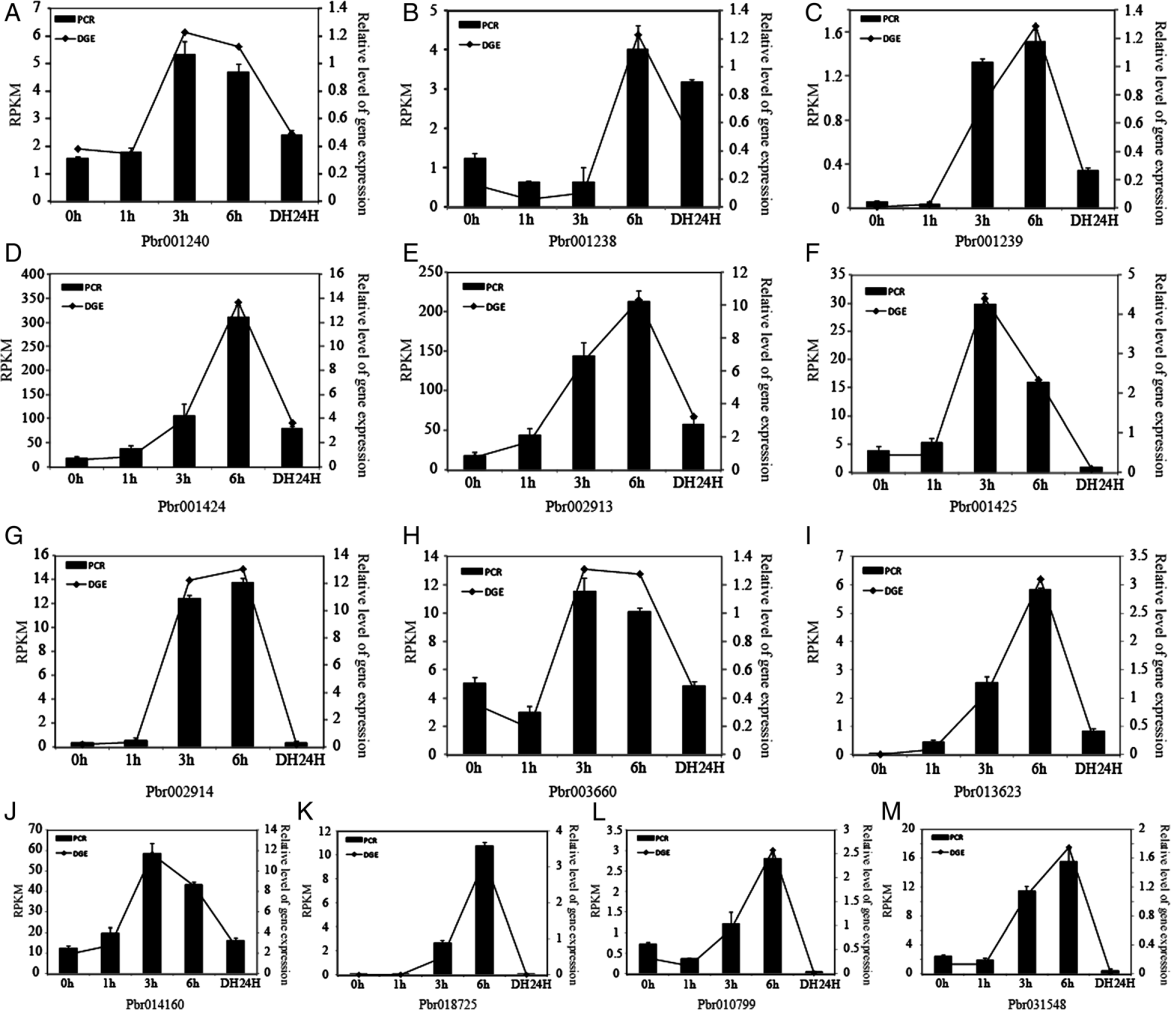
Quantitative RT-PCR analysis of *PbWRKY* gene expression in response to drought stress. D0, D1, D3, D6 and D24, dehydrated for 0, 1, 3 and 6 h in an ambient environment and recovered for 24 h in water, respectively

To validate the expression patterns of the 13 genes in Group 3 in the phylogenetic tree, we performed a qRT-PCR experiment on pear seedlings involving short-term drought stress. The result of qRT-PCR was highly consistent with RNA-seq data (Fig. 5), indicating that our RNA-seq data were reliable. Gene expression levels of all 13 genes increased to their highest level at either 3 or 6 h and then decreased by 24 h of recovery. Twelve genes exhibited the highest level at 6 h after drought stress treatment; and Pbr001425 showed the highest expression level at 3 h of drought treatment.

### Detection of positive selection in Group 2A and subfamily Group 3 *WRKY* genes

Our initial interest in the WRKY proteins came from the proposed role of *WRKY* genes in response to drought stress. As indicated, Groups 2a and 3 were involved in the biological pathways in response to drought stress; therefore, we focused on these two groups. Positive selection is one of the major forces in the emergence of new motifs and functions in genes after duplication events. Selection pressure is measured by ω and the ratio of non-synonymous site (Ka) to synonymous substitution site (Ks), and if a proportion of sites in the sequence provides statistically significant support for ω > 1 along the lineages of interest, then episodic positive selection is inferred. Adaptive evolution frequently occurs in a few sites in a gene, and to a small number of lineages in a phylogenetic tree. In this study, branch-site random effects likelihood (REL) were implemented in Datamonkey [39] to test for positive selection of the *WRKY* genes within Groups 2a and 3. The results identified no WRKYs with positive selection in Group 2a. For branches in Group 3, we found one branch (Pbr001425) under episodic diversifying selection with corrected p-value < 0.0001. The ω value inferred from positively selected sites (ω^+^) along the branch of Pbr001425 was 1187.8, and the proportion of sites evolving at ω^+^ was 7.0 %. To validate the results from Datamonkey, we also performed analysis of positive selection using the branch-site model in PAML for Group 3. The branches being tested for positive selection are referred to as foreground branches, and all other branches on the phylogenetic tree are referred to as background branches. The branch of Pbr001425 was independently defined as a foreground branch. We used branch-site model A (model = 2, NSsites = 2, fix_omega = 0, omega = 1.5) as the alternative hypothesis. The model assumes four classes of sites: class 0 includes codons that are conserved throughout the tree, with 0 < ω_0_ < 1 estimated; class 1 includes codons that are evolving neutrally throughout the tree with ω_1_ < 1; and classes 2a and 2b include codons that are conserved or neutral on the background branches, but become under positive selection on the foreground branches with ω_2_ > 1. The null hypothesis is the branch-site model A, but with ω_2_ = 1 fixed. This null model allows sites to evolve under negative selection on the background lineages and to evolve neutrally on the foreground lineages. Significant positive selection was detected under the χ^2^ test (*p* < 0.01) (Table 3). The results from the two independent types of software indicated that Pbr001425 had undergone positive selection after gene duplication.

**Table 3:** Parameters estimation and likelihood ratio tests for the branch-site models

Hypothesis	lnL	Site class 0	Site class 1	Site class 2a	Site class 2b	2ΔlnL
Alternative	−9212.750	p0 = 0.36828 ω0(b)^1^ = 0.17271 ω0(f)^2^ = 0.17271	p1 = 0.56715	p2a = 0.02542	p2b = 0.03915	35.237**
			ω1(b) = 1	ω2a(b) = 0.17271	ω2b(b) = 1	
			ω1(f) = 1	ω2a(f) = 999	ω2b(f) = 999	
Null	−9230.368	p0 = 0.31965	p1 = 0.45639	p2a = 0.09225	p2b = 0.13171	
		ω0(b=)0.17495	ω1(b) = 1.00000	ω2a(b) = 0.17495	ω2b(b) = 1.00000	
		ω0(f) = 0.17495	ω1(f) = 1.00000	ω2a(f) = 1.00000	ω2b(f) = 1.00000	

Note: **p < 0.01 (χ^2^ test); 1 Background ω; 2 Foreground ω

## Discussion

In this study, a total of 103 *PbWRKY* genes were identified through genome-wide analysis. We adopted the classification scheme for the WRKY family of Eulgem et al. [3]. The *PbWRKY* genes were divided into three distinct clusters: Groups 1, 2 and 3. The Group 2 proteins were further divided into five distinct groups: a–e. However, the phylogenetic tree of *PbWRKY* genes clearly showed that Groups 2a and 2b, and Groups 2d and 2e seemed to form monophyletic clades, respectively. The motifs and exon/intron analysis indicated that Groups 2a, 2b, 2d and 2e were four distinct groups; whereas, Groups 2a and 2b, and Groups 2d and 2e had close phylogenetic relationships, respectively. Additionally, Group 2 was divided into five distinct groups (a–e) with good support values, except for Group 2c. However, the NJ, MP and ML trees consistently clustered Group 2c as a natural clade, supporting the classification of this group. Interestingly, for genes in Group 1, the C-terminal WRKY domain (CTWD) contained one intron, whereas the N-terminal WRKY domain (NTWD) had no introns. The pattern of intron number of the WRKY domain indicated that CTWDs in Group 1 could be ancestors of the WRKY genes in other groups, consistent with the phylogenetic analysis [40].

Gene duplication and divergence events have generally been viewed as a necessary source of evolutionary momentum [20, 21]. In our study, we found that a large fraction of WRKY TFs had arisen by either tandem or segmental duplication (Tables 1 and 2), consistent with results in grapevine [41]. The microsynteny analysis indicated that 33 (32 %) of *PbWRKY* genes were tandemly duplicated and 57 genes (55.3 %) were segmentally duplicated, implying low tandem and high segmental duplications in *PbWRKY* genes, consistent with results for both *Arabidopsis* and grapevine [41, 42]. Compared to tandem duplication, genes arising through segmental duplication may be detected more often in the genome due to sub-functionalization [43, 44]. Genes within a single genome can be classified as singletons, dispersed duplicates, proximal duplicates, tandem duplicates and segmental/WGD [45]. The expression of *PbWRKY* genes in response to drought stress was investigated using RNA-seq data and qRT-PCR. Overall, we found that 44 *PbWRKY* genes were up-regulated at least two-fold under drought treatment, and *PbWRKY* genes in different groups were induced by water deficit treatment, and Groups 2a and 3 were mainly involved in the biological pathways responding to drought stress. All members of Group 2a were up-regulated in response to drought stress. The adaptive evolution analysis showed that no *WRKY*s within Group 2a experienced positive selection, and so the drought stress-related function in Group 2a is highly conservative. In Group 3, 13 *WRKY* genes were induced under drought stress. Most *PbWRKY* genes were induced by drought stress with a peak of expression at 6 h. However, Pbr001425 was mainly up-regulated after 3 h of drought treatment and then expression level decreased after 6 h. Pbr001425 and Pbr001424 were tandemly duplicated genes; however, they had different expression patterns. Furthermore, significant positive selection was detected for Pbr001425. Therefore, we proposed that Pbr001425 underwent positive selection after gene duplication and obtained new functions during evolution.

As an abiotic stress, drought can cause loss of yield and quality of fruit trees [46–48]. In our study, we found 44 drought-responsive *WRKY* genes (Fig. 4). In *Arabidopsis*, four *WRKY* genes were reported to regulate drought response. *AtWRKY57* can elevate ABA levels and so improve drought tolerance of *Arabidopsis* [25]. The grapevine *VvWRKY11* is involved in the response to dehydration stress. Overexpression of *VvWRKY11* in *Arabidopsis* led to more tolerance to water stress induced by mannitol than wild-type plants [49]. Similar to *VvWRKY11*, transgenic *Arabidopsis* lines overexpressing soybean *GsWRKY20* also showed enhanced drought tolerance. Exposure to drought or salt stress triggers many common reactions in plants, such as cellular dehydration, which can lead to osmotic stress and the production of reactive oxygen species [50]. WRKY54 and WRKY70 regulate osmotic stress by working as negative regulators of stomata closure. The *wrky54wrky70* double mutants exhibited clearly enhanced tolerance to osmotic stress [51]. We found that some PbWRKY TFs—orthologs of WRKY TFs that involved in drought, salt and osmotic stresses in *Arabidopsis*—were also responsive to drought stress in pear (Additional file 5), indicating that these PbWRKY TFs may have equivalent functions in pear compared to *Arabidopsis*. However, we also found that some of the orthologous PbWRKY TFs did not respond to drought stress and two of these *Arabidopsis* WRKY TFs did not have orthologous PbWRKY TFs in pear, indicating divergence of WRKY TFs between *Arabidopsis* and pear.

## Methods

### Gene identification

The complete genome, proteome sequences and GFF (General Feature Format) of *Arabidopsis* and pear downloaded from The *Arabidopsis* Information Resource (version 10; http://www.arabidopsis.org) and http://peargenome.njau.edu.cn, respectively. In proteome datasets, if two or more protein sequences at the same locus were identical where they overlapped, we selected the longest sequence. HMMER is used to search sequence databases for WRKY protein sequences. HMMER implements methods using probabilistic models called profile hidden Markov models (profile HMMs). A HMM profile for the WRKY domain (PF03106) was downloaded from the Pfam protein family database (http://pfam.sanger.ac.uk/). HMMER [52] was used to search a customized database containing the proteome with the threshold set of the Pfam GA gathering cutoff. The HMMER-selected proteins were used for a BLASTP query of the original protein database. Finally, the BLASTP hits were scanned for WRKY domains using InterProScan [53]. To confirm our data set of amino acid sequences as WRKYs, we manually examined the conserved amino acid sequence WRKYGQK at the N-terminal and the zinc-finger-like motif at the C-terminal region of the predicted WRKY domain. After removing truncated and pseudo genes, a total of 103 *WRKY* genes were assigned in pear. The CDS and protein sequences of these WRKY genes were stored in Additional files 7 and 8, respectively. These *WRKY* genes were named *PbWRKY* (*Pyrus bretschneideri WRKY*) genes and each given a number designation of 1–73 based on their E-value of InterProScan search in the order of increasing values. The nomenclature and corresponding information are listed in Additional file 1.

### Three building and gene structure prediction

The starting point for our tree construction was the amino acid multiple sequence alignment created using MUSCLE [54] with the default parameters. The Jones, Taylor and Thorton (JTT) with an estimated γ-distribution parameter (G) was selected as the best-fitting amino acid substitution model with four categories using the Akaike information criterion implemented in Model Generator version 85 [55]. The ML analyses were performed using PHYML 3.0 [56], using the JTT + I + G model. Heterogeneity of amino acid substitution rates was corrected using a γ-distribution with five categories. Tree topology searching was optimized using the subtree pruning and regrafting option. The statistical support of the retrieved topology was assessed using a bootstrap analysis with 100 replicates. NJ and MP were implemented with MEGA 5.0 [57]. In NJ and MP, the ‘pairwise deletion’ setting was used. A bootstrap analysis with 1000 replicates was performed in each case. The conserved motifs in the proteins were detected by MEME (http://meme.nbcr.net/meme/cgibin/meme.cgi), with the following parameters: number of repetitions: any; maximum number of motifs: 20; and the optimum motif widths: 6–200 amino acid residues.

### Chromosomal distribution and gene duplication

The genes were plotted separately onto the chromosomes according to gene location in the chromosome in the GFF file using a programmed Perl script. Genes within a maximum of 10 genes distance were considered to be tandem duplicates. The microsyntenies between each pair of members were detected using MicroSyn software [23]. The parameters were set as follows: window size of 50 genes, tandem gap value of 2, expected threshold value cut off of 0.01, and three homologous pairs to define a syntenic segment. Type of gene duplication was determined using the software MCScanX [58].

### Adaptive evolution analysis

Episodic diversifying selection was performed on the Datamonkey web server (http://www.datamonkey.org/) [39], implementing a Branch-site REL approach [59]. Positive selection was validated using the CODEML program contained in the PAML 4 software package [60], using the branch-site model A.

### Data analysis of Solexa/ Illumina sequencing

RNA-seq data of short-term dehydration stress on pear seedlings were obtained from parallel work (paper in preparation) aiming to study the response of pear to water-deficiency stress. Briefly, differentially expressed genes (DEGs) of *Pyrus bretschneideri* were determined using Solexa/Illumina sequencing. The total RNA was extracted from leaves sampled from the seedlings dehydrated for 0, 3, 6 and 24 h of recovery. The library products were ready for sequencing via Illumina HiSeqTM 2000 or other sequencers when necessary. The high-quality clean sequence reads were mapped onto the pear reference genome (http://peargenome.njau.edu.cn) to identify continuous gene regions using SOAP2 [61] and allowed no more than 2-nt mismatching. The unique mapped reads were used for further analysis. For gene expression level analysis, the number of unique-match reads was calculated and then normalized to RPKM (reads per kb per million reads). The gene expression levels were expressed as log(x/y), where x is the detection signal of the treatment sample and y is that of control or the mean of samples. Data were analyzed using Bioconductor.

Genes with similar expression patterns are usually functionally related. We performed a co-expression cluster analysis on the gene expression patterns using cluster software [62] (Additional file 9). In the gene expression profiling analysis, InterPro domains [63] were annotated using InterProScan Release 36.0 [64] and functional assignments were mapped onto Gene Ontology (GO) [65]. The GO classifications and GO enrichment were done using WEGO (Additional files 10 and 11) [66]. For the pathway enrichment analysis, genes in each cluster were mapped to terms in the Kyoto Encyclopedia of Genes and Genomes database (KEGG, release [67]) using BLASTX [68] at E values ≤ 1e-10 to identify significantly enriched KEGG terms. A Perl script was used to retrieve KO (KEGG Ontology) information from the BLAST search result so that pathway associations between unigenes and the database could be established (Additional file 12).

Heat maps were generated using the R package ‘gplots’ (http://www.bioconductor.org/). Clustering in the heat map was carried out using Hierarchical Clustering with the hclust function in R (http://www.r-project.org/).

### Identification of orthologous genes between *Arabidopsis* and pear

To identify orthologous genes between *Arabidopsis* and pear, InParanoid was used with default settings [35]. During InParanoid analysis, an orthology group is initially composed of two so-called seed orthologs that are found by two-way best hits between two proteomes [35]. More sequences are added to the group if there are sequences in the two proteomes that are closer to the corresponding seed ortholog than to any sequence in the other proteome. These members of an orthology group are called inparalogs. In total, there were 118,087 orthology groups identified between *Arabidopsis* and pear, and included 22,580 pear and 15,988 *Arabidopsis* genes. The orthology groups with WRKY TFs involved in drought, salt and osmotic stresses were then extracted.

### Gene expression analysis by qRT-PCR

RNA samples were used for cDNA synthesis using the ReverTra Ace-α First Strand cDNA Synthesis Kit (TOYOBO, TOYOBO Biotech Co. Ltd, Japan) following the manufacturer’s instructions. Primers were designed using Primer5 software based on the target genes (Additional file 13). The 10 μL qPCR solutions contained 5 μL of using SYBR® Green Premix kit (TaKaRa Biotechnology. Dalian, China), 0.25 μM forward and 0.25 μM reverse primer, and 50 ng cDNA templates. The quadruple qRT-PCR reactions were performed on an Lightcycle-480 (Roche) using the following cycling regime: 50 °C/2 min, 95 °C/10 min, followed by 40 cycles of 95 °C/15 s, and 58 °C/1 min. Relative expression levels of each gene were calculated using the 2^-ΔΔCt^ algorithm by normalizing to expression of the pear tubulin gene (AB239681) [69], which was used as an internal control. Four technical replicates were used for each sample and the data are shown as means ± standard errors (SE) (*n* = 3). The source of variation resulted from technical errors, such as operational approach, equipment and reagent. The biological replicates were repeated three times for consistent results, the data were analyzed using analysis of variance (ANOVA) by SAS software (version 8.0, SAS Institute, NC, USA), and statistical differences were compared based on Fisher’s LSD test. The primer sequences used for qRT-PCR are listed in Additional file 13.

## Conclusions

Genome-wide identification, evolutionary analysis, gene structure analysis and expression analysis of pear *WRKY* genes provide us a deep insight of this TF family and their potential roles in drought stress response. This will facilitate the further research on the biological functions of *WRKY* TFs in pear.

## Additional files

Additional file 1:
**List of PbWRKY genes.** (XLS 1392 kb) Additional file 2:
**Phylogenetic tree generated using the Maximum Likelihood (ML) method.** (PDF 169 kb) Additional file 3:
**Phylogenetic tree generated using the Maximum Parsimony (MP) method.** (PDF 186 kb) Additional file 4:
**Consensus sequences of motifs in PbWRKY TFs detected by MEME.** (DOCX 16 kb) Additional file 5:
**List of othology groups with** ***Arabidopsis*** **WRKY TFs that involved in drought, salt or osmotic stress.** (DOCX 26 kb) Additional file 6:
**List of drought responsive PbWRKY TFs that located in co-expression clusters.** (XLS 31 kb) Additional file 7:
**CDS of PbWRKY genes.** (GZ 13907 kb) Additional file 8:
**Protein sequences of PbWRKY TFs.** (TXT 42 kb) Additional file 9:
**List of genes in co-expression gene clusters.** (XLS 1128 kb) Additional file 10:
**GO enrichment analysis of co-expression gene clusters.** (XLS 39 kb) Additional file 11:
**List of genes in enriched GO terms.** (XLS 290 kb) Additional file 12:
**KEGG enrichment analysis of co-expression gene clusters.** (XLS 50 kb) Additional file 13:
**Quantitative RT-PCR analysis of the PbWRKY gene expression in response to drought stress.** (DOCX 17 kb)
