# Supplementary figures and images for: Genome-wide analysis of WRKY transcription factors in white pear (Pyrus bretschneideri) reveals evolution and patterns under drought stress
Source: BMC Genomics. 2015 Dec 24;16:1104. doi: 10.1186/s12864-015-2233-6 (PMC4691019; doi:10.1186/s12864-015-2233-6)

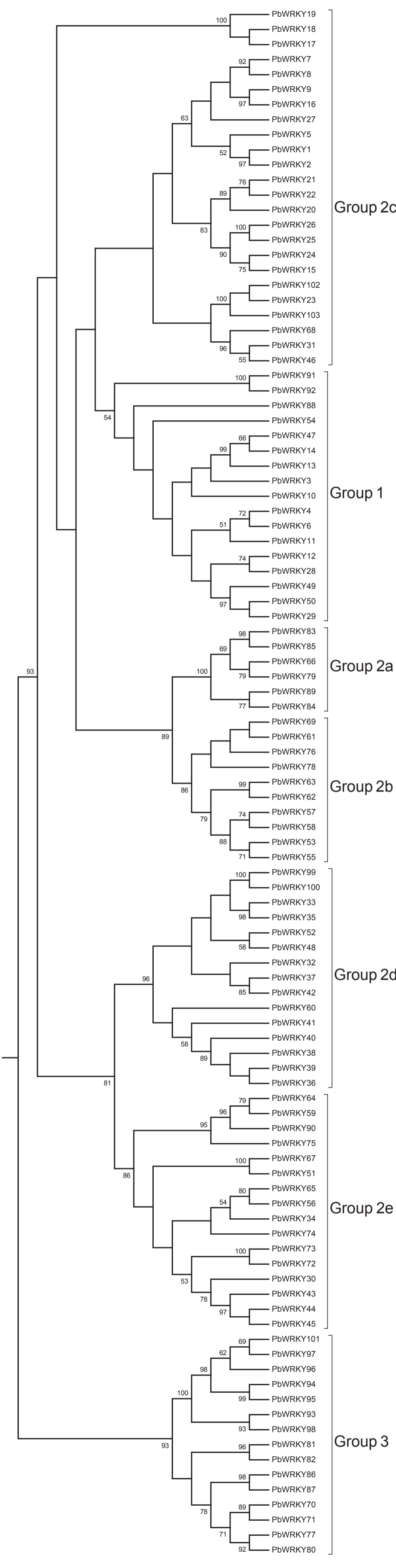

Supplement: Additional file 2: — Phylogenetic tree generated using the Maximum Likelihood (ML) method. (PDF 169 kb) [file 12864_2015_2233_MOESM2_ESM.pdf]

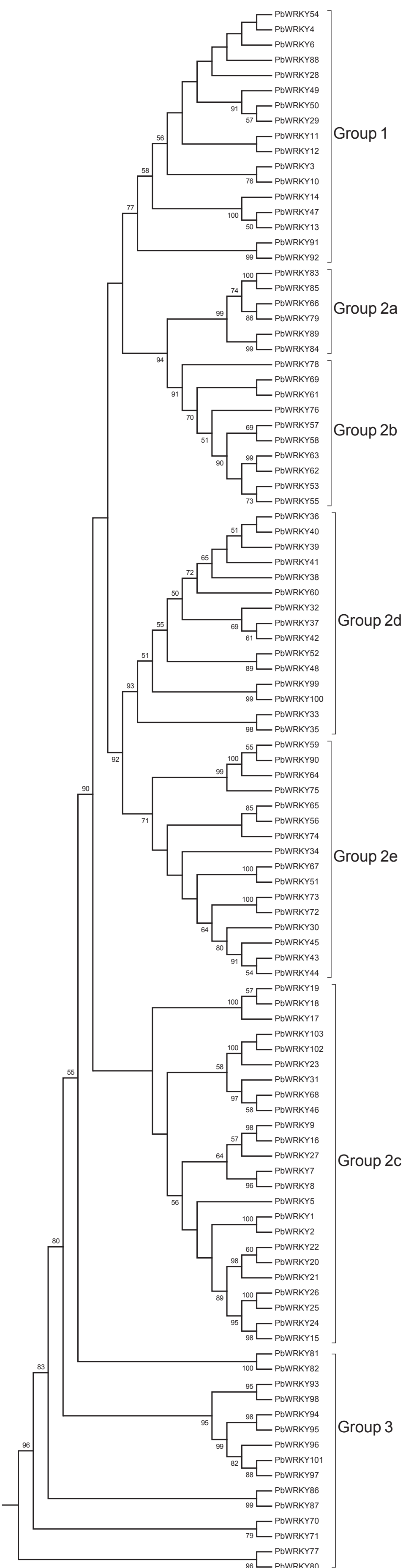

Supplement: Additional file 3: — Phylogenetic tree generated using the Maximum Parsimony (MP) method. (PDF 186 kb) [file 12864_2015_2233_MOESM3_ESM.pdf]
