# Supplementary material for: Genome-wide analysis of WRKY transcription factors in white pear (Pyrus bretschneideri) reveals evolution and patterns under drought stress
Source: BMC Genomics. 2015 Dec 24;16:1104. doi: 10.1186/s12864-015-2233-6 (PMC4691019; doi:10.1186/s12864-015-2233-6)
Supplement: Additional file 4: — Consensus sequences of motifs in PbWRKY TFs detected by MEME. (DOCX 16 kb) [file 12864_2015_2233_MOESM4_ESM.docx]

| **Motif ID** | **Motif consensus sequences** |
| --- | --- |
| Motif 1 | IPSDGYAWRKYGQKPIKGSPYPRGYYRCS |
| Motif 2 | DILDDGYRWRKYGQKVVKGNPCPRSYYRCT |
| Motif 3 | SSKGCPARKHVERSxEDPTILIVTYEGEHNHPLPTARNSLA |
| Motif 4 | GCPVKKQVERSSEDPSIVITTYEGKHNHD |
| Motif 5 | HPNCPVKKKVERSHDGQITEIVYKGEHNH |
| Motif 6 | SDILDDGYRWRKYGQKVVKGNPYPRSYYK |
| Motif 7 | MTLELMNFPKMEDQKAIQEAASQGLQSMEHLIRFLSHQQQHPNNQSARLDCTDITDHTVSKFKKVISLLNRTGHARFRRGPVQPIQPVHFPSSDPLPSQT |
| Motif 8 | KxVREPRVAVQTRSE |
| Motif 9 | KRRKNQVKKVIRVPA |
| Motif 10 | ELAVLQAELQRMNVENQRLREMLTQLTTNYNALQMHLLELMQxQKNQKN |
| Motif 11 | VAAATSAITADPNFTAALAAAITSIIGNG |
| Motif 12 | RELARQLQIHLNAPSSSHGTRELLVQKIILSYEKALSILNSIGSASGCEQQQPTGHVAIRMVESPPHSLNESPRSEDSDREFKDHDNKDSSGKRKNLPRW |
| Motif 13 | EEPVVAGRSTTVESSCKEEKGSPLITDEEDELLGICDSVVSDDFFEGLDGLAEDYFSDHSPVSFGMPWISSSAATAAGSI |
| Motif 14 | LNLPEGLRVISEGLDIREELFPSFNNPSPLNNNYLGSFSPPFAGPTTSGTNYFSMSQQDFGADQNFQSGDIISAATSAANSPSVGLDFPFGQADQLFPNF |
| Motif 15 | GGGGADSSSWDANHDHAAAIKSEDYTEEEISCKSTSTFKDGRGSYKRRKTSHSWTRD |
| Motif 16 | ELVLDCTNPRDSSKFISFDNANCLTNKQEHPFFASFASSSVKEELIVK |
| Motif 17 | ANKVNSDNSLDKENEEFDDDDEEMDETEYGDEDEDDDVVIPDMDMSDEIFMGQKELGSTSS |
| Motif 18 | NMKAEPSPTTGSFLKPQMVYGSLSSTTYSATTVCSDFNTSDERNSGSFEFKPHARSNMVTTDYNHYGNEQPLQIQGQAQPQSHMSPPLAKNEMAVSSNE |
| Motif 19 | GAKPPLSTAPFKKRCHEHDQSDDTSCKFSGSGSASGSGKCH |
| Motif 20 | ATASLNISGFHSNPAAASFSSFDQTSQQQQLLFCLPLSDPIIKPKSAIEELHDLYRPFYPKSQPPLSSPQITPPTLSPLT |

**Table:** **Consensus sequences of motifs in PbWRKYs detected by MEME**
