# Supplementary material for: Genome-wide analysis of WRKY transcription factors in white pear (Pyrus bretschneideri) reveals evolution and patterns under drought stress
Source: BMC Genomics. 2015 Dec 24;16:1104. doi: 10.1186/s12864-015-2233-6 (PMC4691019; doi:10.1186/s12864-015-2233-6)
Supplement: Additional file 5: — List of othology groups with Arabidopsis WRKY TFs that involved in drought, salt or osmotic stress. (DOCX 26 kb) [file 12864_2015_2233_MOESM5_ESM.docx]

| **Gene name** | **Gene Model** | **Description in literature** | **References** | **Orthologous genes in pear** |
| --- | --- | --- | --- | --- |
| ABO3, WRKY63 | AT1G66600 | A WRKY transcription factor in plant responses to ABA and drought stress. | [1] | Pbr001240, Pbr001239, Pbr001238, Pbr001243, Pbr014651, Pbr014648, Pbr001241 |
| WRKY57 | AT1G69310 | Activated expression of WRKY57 confers drought tolerance in Arabidopsis. | [2] | Pbr029950 |
| WRKY53 | AT4G23810 | Activated expression of AtWRKY53 negatively regulates drought tolerance by mediating stomatal movement | [3] | Pbr018725, Pbr013623, Pbr010799, Pbr031548, Pbr003660, Pbr014160 |
| WRKY15 | AT2G23320 | Negative regulator of salt- and osmotic-stress tolerance | [4] | NA |
| WRKY46 | AT2G46400 | Contribute to the feedforward inhibition of osmotic/salt stress-dependent LR inhibition. | [5, 6] | NA |
| WRKY28 | AT4G18170 | Transgenic lines exhibited enhanced tolerance to NaCl, mannitol, and oxidative stress. | [7] | Pbr039547,Pbr021316,Pbr002398 |
| WRKY70 | AT3G56400 | Modulate osmotic stress tolerance by regulating stomatal aperture | [8] | Pbr001424,Pbr002913 |
| WRKY54 | AT2G40750 | Modulate osmotic stress tolerance by regulating stomatal aperture | [8] | Pbr001424,Pbr002913 |
| WRKY25 | AT1G74710 | Increased salt tolerance when overexpressed | [9] | Pbr011477 |
| WRKY33 | AT2G38470 | Increased salt tolerance when overexpressed | [9] | Pbr013092,Pbr011544,Pbr034115,Pbr015939 |
| WRKY40 | AT1G80840 | Modulate the expression of stress-responsive nuclear genes encoding mitochondrial and chloroplast proteins | [10, 11] | Pbr004885,Pbr022408 |
| WRKY18 | AT4G31800 | Modulate the expression of stress-responsive nuclear genes encoding mitochondrial and chloroplast proteins | [10, 11] | NA |
| WRKY60 | AT2G25000 | A direct target gene of WRKY18 and WRKY40 in ABA signaling. | [10] | NA |

Note: *PbWRKY* TFs that were shown in grey background were drought responsive genes.

**References**

1. Ren X, Chen Z, Liu Y, Zhang H, Zhang M, Liu Q, Hong X, Zhu J-K, Gong Z: **ABO3, a WRKY transcription factor, mediates plant responses to abscisic acid and drought tolerance in Arabidopsis**. *Plant J* 2010, **63**:417–429.

2. Jiang Y, Liang G, Yu D: **Activated expression of WRKY57 confers drought tolerance in Arabidopsis.** *Mol Plant* 2012, **5**:1375–88.

3. Sun Y, Yu D: **Activated expression of AtWRKY53 negatively regulates drought tolerance by mediating stomatal movement**. *Plant Cell Rep* 2015:1295–1306.

4. Vanderauwera S, Vandenbroucke K, Inzé A, van de Cotte B, Mühlenbock P, De Rycke R, Naouar N, Van Gaever T, Van Montagu MCE, Van Breusegem F: **AtWRKY15 perturbation abolishes the mitochondrial stress response that steers osmotic stress tolerance in Arabidopsis.** *Proc Natl Acad Sci U S A* 2012, **109**:20113–8.

5. Ding ZJ, Yan JY, Xu XY, Yu DQ, Li GX, Zhang SQ, Zheng SJ: **Transcription factor WRKY46 regulates osmotic stress responses and stomatal movement independently in Arabidopsis**. *Plant J* 2014, **79**:13–27.

6. Ding ZJ, Yan JY, Li CX, Li GX, Wu YR, Zheng SJ: **Transcription factor WRKY46 modulates the development of Arabidopsis lateral roots in osmotic/salt stress conditions via regulation of ABA signaling and auxin homeostasis**. *Plant J* 2015:n/a–n/a.

7. Babitha KC, Ramu S V, Pruthvi V, Mahesh P, Nataraja KN, Udayakumar M: **Co-expression of AtbHLH17 and AtWRKY28 confers resistance to abiotic stress in Arabidopsis.** *Transgenic Res* 2013, **22**:327–41.

8. Li J, Besseau S, Törönen P, Sipari N, Kollist H, Holm L, Palva ET: **Defense-related transcription factors WRKY70 and WRKY54 modulate osmotic stress tolerance by regulating stomatal aperture in Arabidopsis.** *New Phytol* 2013, **200**:457–72.

9. Jiang Y, Deyholos MK: **Functional characterization of Arabidopsis NaCl-inducible WRKY25 and WRKY33 transcription factors in abiotic stresses.** *Plant Mol Biol* 2009, **69**:91–105.

10. Chen H, Lai Z, Shi J, Xiao Y, Chen Z, Xu X: **Roles of arabidopsis WRKY18, WRKY40 and WRKY60 transcription factors in plant responses to abscisic acid and abiotic stress.** *BMC Plant Biol* 2010, **10**:281.

11. Van Aken O, Zhang B, Law S, Narsai R, Whelan J: **AtWRKY40 and AtWRKY63 modulate the expression of stress-responsive nuclear genes encoding mitochondrial and chloroplast proteins.** *Plant Physiol* 2013, **162**:254–71.
