# Supplementary material for: Genome-wide analysis of WRKY transcription factors in white pear (Pyrus bretschneideri) reveals evolution and patterns under drought stress
Source: BMC Genomics. 2015 Dec 24;16:1104. doi: 10.1186/s12864-015-2233-6 (PMC4691019; doi:10.1186/s12864-015-2233-6)
Supplement: Additional file 13: — Quantitative RT-PCR analysis of the PbWRKY gene expression in response to drought stress. (DOCX 17 kb) [file 12864_2015_2233_MOESM13_ESM.docx]

**Quantitative RT-PCR analysis of the *PbWRKY* gene expression in response to drought stress.**

The expression abundance of thirteen selected genes using *Tubulin* as reference gene was analyzed by quantitative real-time PCR. The primers are as following:

| **Genes** | **Primers** | **Sequences (**5’-3’**)** | |
| --- | --- | --- | --- |
|  |  | **Forward** | **Reverse** |
| Pbr001240 | GSP1 | TGGTGGTGGTAGTGGTGTTG | CCGCAGATGATGAATAAGCA |
| Pbr001238 | GSP2 | AATACGATCAAGCGTGCAAA | GGGCGCCATAATATGTTGTT |
| Pbr001239 | GSP3 | CACAACAACCATTCGTCACC | TTAAGCCTCGGCAATCATCT |
| Pbr001424 | GSP4 | ATTTGACCAAGGTTGCAAGG | CTCGCCGATGTAGGTGATTT |
| Pbr002913 | GSP5 | TGAGATCGCCTAGGATCAGG | TGAGATCGCCTAGGATCAGG |
| Pbr001425 | GSP6 | CCAGCACCTAGGATTGGAAA | CCTCTGGACTTGCTTCTTGG |
| Pbr002914 | GSP7 | CAGCAACCACTGAGTCTCCA | CACAACTGCCACCGATCATA |
| Pbr003660  Pbr014160  Pbr013623  Pbr018725  Pbr010799  Pbr031548 | GSP8  GSP9  GSP10  GSP11  GSP12  GSP13 | ATTACTTTGCGCTGCCTAGC  AATGAGCAACGTTCGGAATC  ACAGGTCATGTTGCAATTCG  AGGCTACTACCGCTGCACTC  TGGGAGCAAAGGAGTCTTGT  CTCTATCGGCTCACCCTCAG | GGAATCGGAGAATTGGTTGA  ACTGGCTGCAGTGGAAAATC  GCGGTCAGAGTCTTCACTCC  TCAAAGATCGTCGGGTCTTC  GATGGAGCGTTGAGATGGAT  TTGTTGTCGTGGTCCTTGAA |
